# The Prosigna gene expression assay and responsiveness to adjuvant cyclophosphamide-based chemotherapy in premenopausal high-risk patients with breast cancer

**DOI:** 10.1186/s13058-018-1012-0

**Published:** 2018-07-27

**Authors:** Maj-Britt Jensen, Anne-Vibeke Lænkholm, Torsten O. Nielsen, Jens Ole Eriksen, Pernille Wehn, Tressa Hood, Namratha Ram, Wesley Buckingham, Sean Ferree, Bent Ejlertsen

**Affiliations:** 1 Danish Breast Cancer Cooperative Group, Rigshospitalet, Copenhagen University Hospital, DBCG Secretariat, Bldg. 2501 Rigshospitalet, Blegdamsvej 9, DK-2100 Copenhagen, Denmark; 2 Department of Surgical Pathology, Zealand University Hospital, Slagelse, Denmark; 3 Department of Pathology and Laboratory Medicine, University of British Columbia, Vancouver, BC Canada; 4 NanoString Technologies, Inc, Seattle, WA USA; 5 Danish Breast Cancer Cooperative Group, Department of Oncology, Rigshospitalet, Copenhagen University Hospital, Copenhagen, Denmark

**Keywords:** Breast neoplasms, Adjuvant chemotherapy, Cyclophosphamide, CMF, PAM50

## Abstract

**Background:**

The PAM50-based (Prosigna) risk of recurrence (ROR) score and intrinsic subtypes are prognostic for women with high-risk breast cancer. We investigate the predictive ability of Prosigna regarding the effectiveness of cyclophosphamide-based adjuvant chemotherapy in premenopausal patients with high-risk breast cancer.

**Methods:**

Prosigna assays were performed on the NanoString platform in tumors from participants in Danish Breast Cancer Group (DBCG) 77B, a four-arm trial that randomized premenopausal women with high-risk early breast cancer to no systemic treatment, levamisole, oral cyclophosphamide (C) or cyclophosphamide, methotrexate and fluorouracil (CMF).

**Results:**

In total, this retrospective analysis included 460 women (40% of the 1146 randomized patients). The continuous Prosigna ROR score was prognostic in the no systemic treatment group (unadjusted *P* < 0.001 for disease-free survival (DFS), *P* = 0.001 for overall survival (OS)). No statistically significant interaction of continuous ROR score and treatment on DFS and OS was found. A highly significant association was observed between intrinsic subtypes and C/CMF treatment for DFS (*P*_interaction_ = 0.003 unadjusted, *P* = 0.001 adjusted) and OS (*P*_interaction_ = 0.04). In the adjusted analysis treatment with C/CMF was associated with a reduced risk of DFS events in patients with basal-like (hazard ratio (HR) 0.14; 95% CI 0.06; 0.32) and luminal B (HR 0.48; 95% CI 0.27; 0.84) subtypes but not in patients with Human epidermal growth factor receptor-enriched (HR 1.05; 95% CI 0.56; 1.95) or luminal A (HR 0.61; 95% CI 0.32; 1.16) subtypes.

**Conclusion:**

The Prosigna ROR score and intrinsic subtypes were prognostic in high-risk premenopausal patients with breast cancer, and intrinsic subtypes identify high-risk patients with or without major benefit from adjuvant C/CMF treatment.

**Electronic supplementary material:**

The online version of this article (10.1186/s13058-018-1012-0) contains supplementary material, which is available to authorized users.

## Background

The early results of the first adjuvant Milan trial were published in 1976 [1], and showed a clear benefit from 4-weekly oral cyclophosphamide on days 1 to 14 combined with intravenous methotrexate and fluorouracil on days 1 and 8 (CMF). The Milan trial included patients with high-risk (node-positive) breast cancer who were randomized after mastectomy; none of whom received radiotherapy or endocrine treatment. Several cooperative groups, including the Danish Breast Cancer Group (DBCG), initiated trials to explore the results from the pivotal Milan trial. DBCG 77B included premenopausal patients with high-risk breast cancer who after mastectomy and radiotherapy and without endocrine treatment were randomized to observation, levamisole, single-agent cyclophosphamide, or CMF. Results showed a similar benefit from oral cyclophosphamide and CMF, and the survival benefit was maintained after 25 years of follow up [2]. Due to the results of these and other early clinical trials, adjuvant chemotherapy has been considered a standard in premenopausal patients with node-positive breast cancer since the National Institutes of Health Consensus Conference in 1980 [3]. Subsequent meta-analyses by the Early Breast Cancer Trialists’ Collaborative Group (EBCTCG) have demonstrated that the relative benefit of CMF was largely independent of patient characteristics, nodal status and other tumor features available for analysis, and also independent of the use of concomitant tamoxifen. Furthermore, in the EBCTCG analysis an additional incremental benefit was shown from adding an anthracycline to CMF, from substituting methotrexate with doxorubicin or epirubicin, and from giving taxanes concurrently or in sequence with anthracyclines [4, 5].

Since intrinsic molecular subtypes were first described more than 15 years ago, their prognostic ability has repeatedly been demonstrated in early breast cancer [6]. However, few attempts have been made to explore the ability of these subtypes to predict benefit of chemotherapy. The prediction analysis of microarray 50 (PAM50) gene set has become a standard for identifying intrinsic subtypes from RNA expression measurements and its predictive abilities have been evaluated in the Canadian MA.5 trial comparing adjuvant cyclophosphamide, epirubicin, and fluoroucil (CEF) to CMF in patients with early breast cancer. The human epidermal growth factor receptor 2 (Her2)-enriched subtype was associated with a benefit from the anthracycline-containing chemotherapy arm while no significant differences between arms were shown for patients with basal-like, luminal A or luminal B breast cancers [7]. Additionally, a high 21-gene recurrence score (OncotypeDx) has been associated with benefit from addition of cyclophosphamide, adriamycin, and fluorouracil (CAF) to tamoxifen [8] in high-risk node-positive disease, suggesting that not all patients with high-risk early breast cancer derive a similar benefit from adjuvant chemotherapy.

The PAM50 gene signature has been developed into a clinical test, the Prosigna gene signature assay, validated to estimate the prognosis for postmenopausal patients with estrogen receptor (ER)+ early-stage breast cancer [9, 10]. In the DBCG 77B trial we randomized premenopausal patients with high-risk breast cancer to cyclophosphamide-based chemotherapy against no systemic treatment, and the two primary co-objectives of this study were to evaluate the predictive ability of the PAM50-based Prosigna risk of recurrence (ROR) score and intrinsic subtypes.

## Methods

Details on DBCG 77B have been published previously [2]. In brief, this was an open-label randomized phase III trial comparing, in the adjuvant setting, radiotherapy alone (control), radiotherapy plus 2.5 mg/kg/body weight of levamisole on 2 days consecutively each week for 48 weeks, radiotherapy plus 12 cycles of single-agent cyclophosphamide (C) 130 mg/m^2^ orally on days 1–14 every 4 weeks, or radiotherapy plus 12 cycles of CMF (cyclophosphamide 80 mg/m^2^ orally on days 1–14, methotrexate 30 mg/m^2^ intravenously on days 1 and 8, and 5-fluorouracil 500 mg/m^2^ intravenously on days 1 and 8) every 4 weeks. Patients were eligible for DBCG 77B if they were without distant metastasis, were premenopausal, and had either positive lymph nodes, tumors > 5 cm, and/or invasion of the deep fascia. The DBCG prepared the original 77B trial and its biological sub-studies have previously been described in detail [2, 11]. The Biomedical Research Ethics of the Danish Capital Region approved the protocol (H-15012740).

### Central assessment of Prosigna

Formalin-fixed, paraffin-embedded tumor blocks (FFPE) from primary excisional surgery specimens were collected at the Department of Pathology, Zealand University Hospital. RNA extraction and Prosigna testing were performed according to standard operating procedures [9] by investigators blinded to clinical outcome. Prosigna results were transferred to the data manager at Nanostring, who while remaining blinded to clinical data, prepared the Prosigna ROR and intrinsic subtype analysis data set. This was forwarded to the DBCG statistical office for merging with clinical data and to execute the prespecified statistical analysis plan. ROR cut offs for analysis of the combined study population (including ER+/− and Her2+/−) were prespecified in terms of the observed ROR score distribution tertiles within the study population (8–51 (low), 52–71 (intermediate), and 72–100 (high)) and were independent of nodal status.

The age of the FFPE tissue blocks resulted in decreased RNA quality compared with previous studies performed by the DBCG. In order to increase power for the exploratory analysis in the ER+/Her2- population, the Prosigna test quality control criteria for RNA quality was relaxed for this subset.

### Statistical methods

A written prespecified statistical analysis plan was finalized prior to data analysis. The statistical analysis was executed by the DBCG statistical office, which was not involved in biomarker data collection. The primary endpoint for this study was disease-free survival (DFS), defined as time from randomization to any first event of invasive ipsilateral or contralateral breast cancer recurrence, local or regional invasive recurrence, distant recurrence, second (non-breast) invasive cancer, or death from any cause. The secondary endpoint was overall survival (OS), defined as time from date of randomization until death from any cause. Survival rates were estimated by the Kaplan-Meier method. Patients treated according to protocol were included. Cox proportional hazards regression models were applied to assess the unadjusted and adjusted hazard ratios (HR). The chemotherapy arms (C + CMF) were analyzed versus no chemotherapy arms (control + levamisole). The multivariate models included age at entry (≤ 40, 41–45, 46–50, and 50 years), tumor size (≤ 2, > 2 to ≤ 5, and > 5 cm), lymph node (LN) status (0–3 positive LN, 4–9, 10+ and < 10 vs 10+ retrieved), histologic type (ductal vs non-ductal), grade (ductal I, II, and III) and treatment regimen. Proportional hazards assumptions were assessed using Schoenfeld residuals and by including a time-dependent component for each covariate. The hazard rates for histologic type and grade were not proportional; therefore, stratification was used. To comply with proportional hazards assumptions about subtypes and ROR score, separate estimates were included according to time since randomization. The Wald test was used to assess heterogeneity. Associations between included and excluded patients and clinico-pathological characteristics (excluding unknowns) were analyzed using the chi-square test. *P* values are two-tailed, unadjusted for number of comparisons. Central review, monitoring, and statistical analyses were done by the DBCG Statistical Office using the SAS 9.4 software program package (SAS Institute, Cary, NC, USA).

## Results

The DBCG 77B trial enrolled 1146 patients, among whom 1072 received treatment as allocated by randomization (Additional file 1: Figure S1). Tumor blocks were available from 649 patients, and 623 blocks contained sufficient invasive breast cancer tissue for RNA extraction. The Prosigna assay was successful in 487 patients, of which 460 were treated according to protocol. The assessable 460 patients did differ significantly from the 612 non-assessable patients (*P* < 0.05) with regard to histologic type (*P* = 0.03) and malignancy grade (*P* = 0.02). Among the 460 patients, 231 (81%) experienced a first event within 10 years. Number of positive lymph nodes, tumor size, age, and treatment regimen were not significantly different in assessable vs non-assessable patients. The treatment effect was similar with an HR favoring chemotherapy for DFS (adjusted HR; 0.55; 95% CI, 0.38 to 0.79; *P* = 0.001) to the effect observed in the original study (HR = 0.60; 95% CI, 0.48 to 0.75). Table 1 shows the baseline characteristics according to Prosigna subtype for the 460 patients: 324 patients were included in the exploratory analysis of DFS in the ER+/Her2− patient population.

**Table 1:** Patient and tumor characteristics by Prosigna (PAM50) subtype

	Total study set		Molecular subtype							
			Luminal A		Luminal B		Basal-like		Her2-E	
Characteristics	*N*	%	*N*	%	*N*	%	*N*	%	*N*	%
Number of patients	460	–	161	35	118	27	61	13	120	26
Age										
< 40 years	96	21	23	14	26	22	19	31	28	23
40–49 years	233	51	94	58	63	53	25	41	51	43
50–59 years	131	28	44	27	29	25	17	28	41	34
Lymph nodes excised										
None	19	4	8	5	7	6	1	2	3	3
1–3	110	24	42	26	27	23	14	23	27	23
4–9	268	58	97	60	67	57	39	64	65	54
> 9	6	14	14	9	17	14	7	11	25	21
Lymph node status										
Negative	43	9	16	10	4	3	10	16	13	11
1–3 positive	271	59	106	66	72	61	33	54	60	50
4+ positive	127	28	31	19	35	30	17	28	44	37
Unknown	19	4	8	5	7	6	1	2	3	3
Tumor size										
0–20 mm	132	29	60	37	36	31	14	23	22	18
21–50 mm	239	52	77	48	61	52	30	49	71	59
> 50 mm	85	18	24	15	21	18	13	21	27	23
Unknown	4	1	0	0	0	0	4	7	0	0
Deep fascia invasion										
Absent	346	75	127	79	93	79	45	74	81	68
Present	109	24	32	20	25	21	15	25	37	31
Unknown	5	1	2	1	0	0	1	2	2	2
Histologic type										
Ductal carcinoma	393	85	132	82	99	84	52	85	110	92
Lobular carcinoma	33	7	16	10	13	11	1	2	3	3
Other	28	6	11	7	5	4	6	10	6	5
Unknown	6	1	2	1	1	1	2	3	1	1
Malignancy grade^a^										
Grade I	65	17	46	35	15	15	1	2	3	3
Grade II	242	62	81	61	73	74	22	42	66	60
Grade III	86	22	5	4	11	11	29	56	41	37
HR status										
Positive	329	72	146	91	112	95	6	10	65	54
Negative	114	25	6	4	5	4	52	85	51	43
Unknown	17	4	9	6	1	1	3	5	4	3
Systemic treatment										
Control or levamisole	113	25	37	23	34	29	10	16	32	27
C or CMF	347	75	124	77	84	71	51	84	88	73
ROR score groups										
Low (0–51)	155	34	135	84	2	2	14	23	4	3
Intermediate (52–71)	148	32	26	16	50	42	40	66	32	27
High (72–100)	157	34	0	0	66	56	7	11	84	70

*PAM50* prediction analysis of microarray 50, *C* cyclophosphamide, *CMF* cyclophosphamide, methotrexate and fluorouracil, *HR* hormone receptor, *Her2-E* human epidermal growth factor receptor 2-enriched, *ROR* risk of recurrence

^a^Ductal carcinomas only

Of the 460 patients included in the primary analyses 61 (13%) were classified as having basal-like, 120 (26%) as Her2-enriched, 161 (35%) as luminal A and 116 (25%) as luminal B breast cancer, reflecting the population including patients with ER+/− and Her2+/− breast cancer.

### Prognosis by Prosigna ROR score

**Fig. 1:**
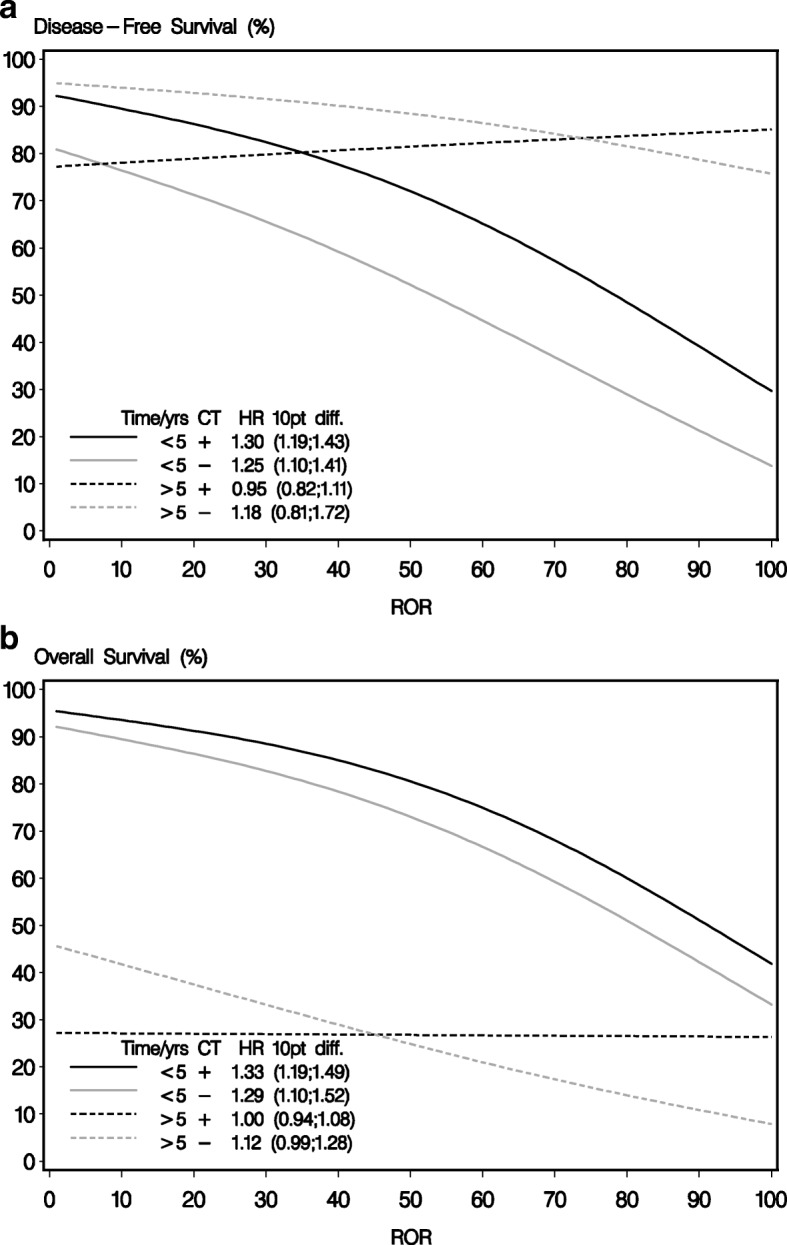
**a** Disease-free survival by continuous risk of recurrence (ROR) score 0–5 years and 5–10 years after randomization, respectively, for patients in the systemically untreated regimen (CT –) and patients in the C/CMF arm (CT +). **b** Overall survival by continuous ROR score for 0–5 years and 5+ years after randomization, respectively. Hazard ratios and corresponding 95% CI for a 10-point difference in continuous ROR score are shown

**Fig. 2:**
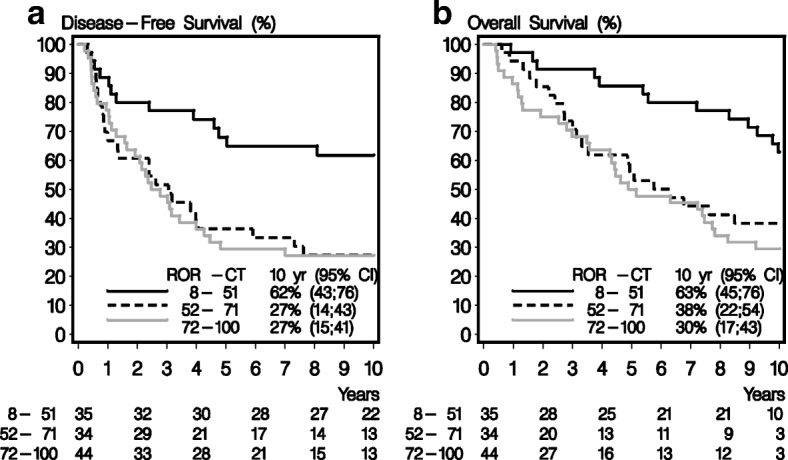
**a** Kaplan-Meier estimates of disease-free survival in the 113 patients systemically untreated (– CT), according to low, intermediate, and high Prosigna risk of recurrence (ROR) scores. **b** Kaplan-Meier estimates of overall survival

The continuous ROR score was highly prognostic (unadjusted HR, 1.23; 95% CI, 1.09 to 1.39, *P* < 0.001 for a 10-point difference) for DFS within the group not treated systemically. For the C/CMF-treated group, the ROR continuous score had a different effect according to follow-up time (0–5 years vs 5+ years) (Fig. 1a and b). The ROR prognosis was statistically significant for the first 5 years for both the C/CMF arm and the no chemotherapy arm, and with no differential effect, whereas after 5 years no prognostic effect was apparent. There was no statistically significant differential effect according to follow-up time in the no chemotherapy arm alone. Including all patients, irrespective of hormone receptor status, the 10-year DFS rates in the untreated group were 62% (95% CI, 43 to 76), 27% (95% CI, 14 to 43) and 27% (95% CI, 15 to 41) for low, intermediate, and high ROR scores, respectively (Fig. 2a). Comparing intermediate ROR with low-risk ROR for the untreated group, unadjusted HR = 2.66; 95% CI, 1.35 to 5.24, and similarly for the high vs low-risk ROR group HR = 2.73; 95% CI, 1.43 to 5.22. Likewise, the ROR score was prognostic for OS in the untreated group (Fig. 2b) and the 10-year OS rates were 63% (95% CI, 45 to 76), 38% (95% CI, 22 to 54), and 30% (95% CI, 17 to 43) for low, intermediate, and high ROR scores, respectively.

### Effect of chemotherapy according to the Prosigna ROR score

**Fig. 3:**
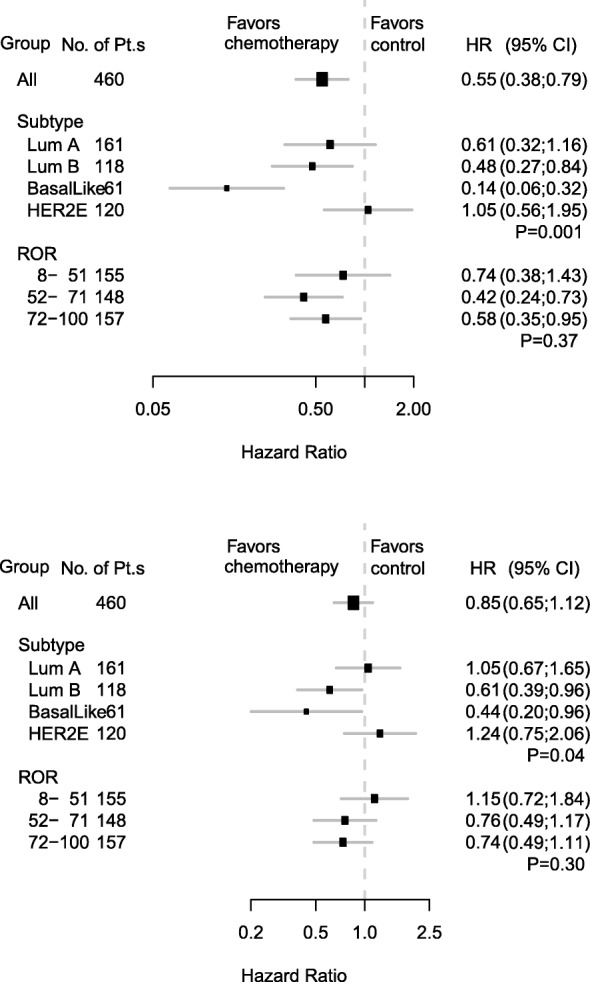
Forest plots illustrate proportional hazard models for disease-free survival (**a**) and overall survival (**b**) overall and according to intrinsic cancer subtype and risk of recurrence (ROR) score, respectively. Hazard ratios refer to adjusted estimates obtained in the multivariate analysis. *P* values are for test of heterogeneity of treatment effect. Boxes represent the weight of data for each subgroup relative to the total data. Pt.s, patients; Lum, luminal; HER2E, human epidermal growth factor receptor 2-enriched

For the first co-primary objective, we examined the association between the continuous Prosigna ROR score and benefit of chemotherapy in the population including patients with ER+/− and Her2+/− breast cancer. There was no significant effect of the ROR score on treatment; for the continuous ROR score *P*_interaction_ = 0.66 for DFS and *P*_interaction_ = 0.32 for OS, in unadjusted models. The benefit for DFS was significant for both intervals of intermediate (52–71) and high (72–100) Prosigna ROR scores, whereas there was a smaller, non-significant benefit for low (0–51) ROR scores, although interactions between ROR score groups and benefit of chemotherapy were not statistically significant (Table 2 for unadjusted results) (*P*_interaction_ = 0.47), also true for OS (P_interaction_ = 0.24). The HR for impact of the continuous ROR as the impact of the predefined intervals remained largely unchanged following adjustment for patients and tumor characteristics (Fig. 3a and b).

**Table 2:** Unadjusted HR estimates of the treatment effect for DFS and OS according to Prosigna ROR scores and subtype

	DFS			OS		
	HR	(95% CI)	P_interaction_	HR	(95% CI)	*P*_interaction_
ROR score			0.47			0.24
Low tertile	0.79	(0.42–1.47)		1.09	(0.70–1.71)	
Intermediate tertile	0.49	(0.30–0.79)		0.75	(0.50–1.13)	
High tertile	0.64	(0.42–0.98)		0.66	(0.46–0.96)	
Subtype			0.003			0.04
Luminal A	0.64	(0.36–1.14)		0.96	(0.62–1.48)	
Luminal B	0.47	(0.28–0.77)		0.51	(0.33–0.77)	
Basal-like	0.19	(0.09–0.40)		0.52	(0.24–1.12)	
Her2-enriched	1.04	(0.62–1.75)		1.10	(0.71–1.71)	

*DFS* disease-free survival, *OS* overall survival, *HR* hazard ratio, *ROR* risk of recurrence, *95% CI* 95% confidence interval, *P*_*interaction*_ *P* derived from a Wald test for heterogeneity, *Her2* human epidermal growth factor receptor 2

Prosigna has been validated and is indicated for use in ER+, Her2− disease only, therefore, an underpowered but planned exploratory analysis of DFS in the ER+, Her2- subset was also executed using the risk groups defined by the commercial cut point of low-risk ROR ≤ 40. A benefit from chemotherapy was observed in the high-risk group (HR = 0.48, 95% CI, 0.33 to 0.69) while no benefit was demonstrated in the low-risk group (HR = 1.13, 95% CI, 0.42 to 3.07); however, no there was no statistically significant interaction between ROR risk groups and treatment effect (unadjusted *P*_interaction_ = 0.11) (Additional file 2: Figure S2) and (adjusted *P*_interaction_ = 0.10).

### Effect of chemotherapy for Prosigna intrinsic subtype groups

**Fig. 4:**
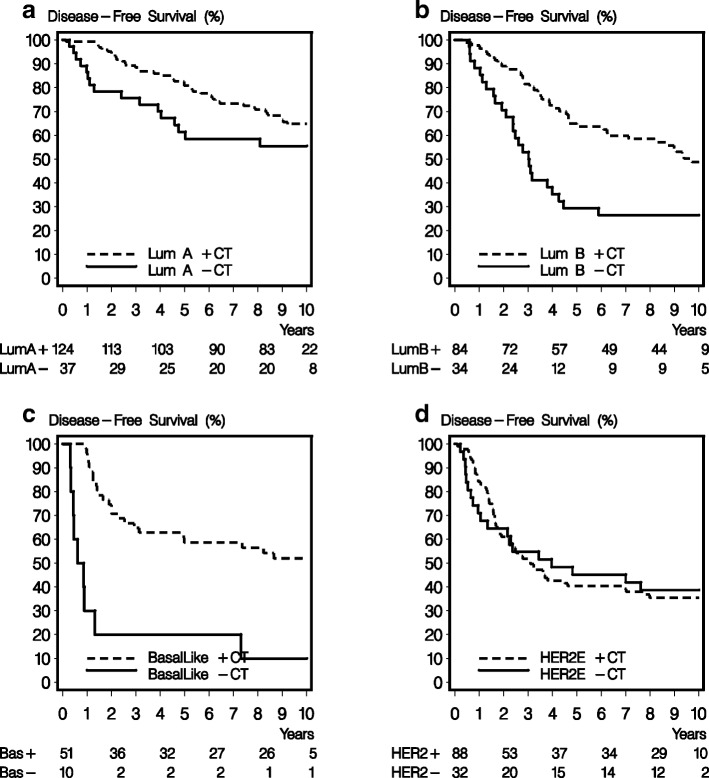
Kaplan-Meier estimates of disease-free survival in patients with luminal (Lum) A (**a**), luminal B (**b**), basal-like (**c**), and human epidermal growth factor receptor 2-enriched (HER2-E) (**d**) breast cancer in the systemically untreated arm (–CT) and in the cyclophosphamide/cyclophosphamide, methotrexate and fluorouracil arm (+CT)

For the second co-primary objective, we examined the association between Prosigna intrinsic subtypes and benefit of chemotherapy. A significant interaction was observed between Prosigna subtype and treatment (Table 2) for DFS (*P*_interaction_ = 0.003) and OS (*P*_interaction_ = 0.04), with the subtypes included separately. Statistically significant interactions between the Prosigna subtypes and treatment remained after multivariate adjustment both for DFS (Fig. 3a) and OS (Fig. 3b). Patients with a basal-like subtype of breast cancer had a pronounced benefit from cyclophosphamide-based adjuvant chemotherapy (unadjusted HR, 0.19; 95% CI, 0.09 to 0.40), and a marked benefit was also obtained by those with the luminal B subtype (unadjusted HR, 0.47; 95% CI, 0.28 to 0.77) (Fig. 4). While there was significant benefit from chemotherapy on DFS in both the subgroups with basal-like and luminal B subtype breast cancer, there was no effect in women with Her2-enriched tumors, and a less pronounced effect in those with luminal A tumors (*P*_adjusted_ = 0.001). The benefit was not significantly different in patients with the luminal A subtype from those with the luminal B subtype (*P*_adjusted_ = 0.52) or from those with non-Luminal A subtype tumors combined (*P*_adjusted_ = 0.87). For OS the corresponding figures are *P*_adjusted_ = 0.09 and *P*_adjusted_ = 0.32.

## Discussion

Premenopausal patients with high-risk breast cancer are generally recommended chemotherapy, but our study suggests that patients with tumors from different intrinsic molecular subtypes do not benefit equally from cyclophosphamide or CMF-based adjuvant chemotherapy. When considering a heterogeneous population of patients with ER+/− and Her2+/− disease, the continuous Prosigna ROR score was shown to be significantly associated with prognosis. The Prosigna ROR score was not significantly predictive of treatment effect in a population combining ER+/− and Her2+/− patients. However, the biology of these disease subtypes is dramatically different and it is not unexpected that a score developed to predict disease recurrence in ER+ Her2− patients does not predict benefit from chemotherapy across all ER+/− and Her2+/− patients. By focusing on the more homogenous population of ER+, Her2− patients we did observe a benefit from chemotherapy in the high-risk population and no benefit in the low-risk population though similarly, we found no statistically significant heterogeneity of treatment effect according to ROR score. This subset analysis was hindered by the limited number of patients included, especially for the group not treated with chemotherapy.

More crucially, a significantly different and clinically relevant benefit was obtained based on the major intrinsic subtypes as assigned by Prosigna. The use of cyclophosphamide-based chemotherapy in patients with basal-like breast cancer was associated with an 86% relative reduction in DFS events and a 56% relative reduction in mortality. Patients with luminal B disease also had a statistically significant benefit from chemotherapy, albeit to a lesser degree (39% reduction in DFS events and in mortality). In contrast, patients with tumors of the Her2-enriched subtype and luminal A subtype obtained no statistically measurable benefit. The gain obtained by patients with a luminal A subtype however, did not significantly differ from those with a luminal B nor from those with a non-luminal A subtype taken together.

This heterogeneity in benefit from chemotherapy according to the intrinsic subtypes explains the lack of association between the ROR score and benefit from chemotherapy in the overall population. Though the tumors with the lowest ROR are the luminal A subtype, with little sensitivity to chemotherapy, a large proportion of patients with basal-like breast cancer were assigned intermediate ROR scores and saw a large benefit from chemotherapy and most of the chemotherapy-insensitive Her2-enriched tumors were assigned high ROR scores. Consequently, within the overall population there is not a monotonic relationship between the ROR score and benefit from chemotherapy. However, when focusing on the ER+/Her2− population, we found a more direct relationship between risk and benefit from chemotherapy, although the analysis in this subset was not powered to show an interaction.

Our results are complementary to the results from the NCIC-CTG MA.5 trial, where all patients were treated with adjuvant chemotherapy but were randomized to CMF or CEF. Cheang and colleagues showed no added benefit in basal-like breast cancer but identified a substantial benefit in Her2-enriched high-risk breast cancer from the anthracycline-containing CEF chemotherapy as compared to CMF [7]. The MA.5 and DBCG 89D trials and a pooled analysis with three additional phase III trials showed a greater benefit of anthracyclines in patients with *TOP2A* alterations and a trend towards greater benefit in patients with Her2-amplified tumors [12–15]. More recently, the DBCG READ trial gave no evidence to support a benefit from anthracyclines in patients with early *TOP2A*-normal breast cancer while the Anthracyclines in early breast cancer (ABC) trials suggested that patients with Her2-normal breast cancer derive some benefit from anthracyclines [16, 17]. The association between the Her2-enriched subtype and alteration of *TOP2A* has not yet been clarified but may improve the clinical utility of both markers. Finally, retrospective analyses of clinical studies in the metastatic setting have shown that the intrinsic subtype may predict benefit from other specific cytotoxic agents such as gemcitabine [18] and docetaxel [19]. Together these studies emphasize that the potential benefit to patients with breast cancer from establishing an association between intrinsic subtype and type of chemotherapy could be significant.

In this subset we showed a distribution of subtypes that differs from that previously demonstrated by immunohistochemical analysis (IHC) using tissue microarrays constructed from 633 patients participating in the DBCG 77B trial [11]. The treatment effect appear less segregated between the luminal A and B subtypes using Prosigna compared to IHC, presumably due to the smaller percentage of luminal A tumors identified by the IHC definition employed in the previous study. In contrast, the effect obtained by an IHC definition of core-basal cancer was less pronounced compared to basal breast cancers as defined by Prosigna [11]. While separate studies have shown that nucleic acid-based methods surpass the analytical reproducibility of IHC, our study confirms that genomic-based tests provide also additional information on clinical benefit compared to IHC surrogates [20, 21].

Due to the very old age of the DBCG77B study, we were only able to collect tumor blocks to be used for messenger (m)RNA testing from 623 of the original 1146 patients, and samples from the no chemotherapy arms (which were closed early and recruited smaller numbers of patients) were particularly sparse (Additional file 1: Figure S1), reducing study power compared to the original [2] and even to subsequent IHC studies [11] for which more cases were available. As a consequence, the ability to conduct subgroup analysis has been limited e.g. the possible influence of chemotherapy-induced amenorrhea, which may have been particularly important in patients with luminal cancers.

Beyond power concerns, this study has other potential limitations. First, the retrospective evaluation of only a subset of patients in the biomarker analysis introduces a risk of bias and a residual risk may persist despite adjustments in the multivariate analyses. Second, the 77B study samples analyzed here had been stored for almost 40 years resulting in significant degradation in the mRNA in the samples. This degradation was evident in the higher rate of samples that were unevaluable by the assay (22%) compared to 1% in a previous study using samples banked for a median of 10 years [22]. DBCG77B only employed cyclophosphamide or classic CMF and benefit of a more pronounced effect could potentially have been obtained by more contemporary adjuvant chemotherapy. The contribution from cyclophosphamide might, however, have been inseparable from the contributions of anthracyclines, taxanes, endocrine therapy, and Her2-targeted therapy, if these treatments were combined. A possible association between molecular tumor subtype and the individual drugs may in later studies be obtained by identifying trials with successive addition of anthracyclines and taxanes.

Strengths of this study include use of patients with high-risk breast cancer derived from a phase III randomized trial without any biomarker selection. The inclusion of a control group who were not systemically treated was crucial for our ability to isolate associations between intrinsic subtypes by Prosigna and treatment effect from cyclophosphamide based-chemotherapy. In addition, our study adhered carefully to the ReMARK guidelines [23], and used the validated and Food and Drug Association (FDA)-cleared Prosigna assay following standard operating procedures as specified by the manufacturer [24].

## Conclusions

In summary, this study demonstrates that the Prosigna assay is both prognostic and predictive of benefit from cyclophosphamide-based adjuvant chemotherapy in premenopausal patients with high-risk breast cancer. These results provide further evidence that the high-risk intrinsic subtypes of breast cancer have highly differential responses to cyclophosphamide-based chemotherapy regimens, with clear benefit in the basal-like and luminal B subtypes.

## Additional files

Additional file 1: **Figure S1.** Consort flow diagram. (PDF 234 kb)

Additional file 2: **Figure S2.** Kaplan-Meier estimates of disease-free survival of the ER+, Her2- population in the systemically untreated regimen (-CT) and in the C/CMF arm (+CT), according to low (≤ 40) and high (> 40) Prosigna ROR scores. (EPS 183 kb)

## Abbreviations

ABC: Anthracyclines in early breast cancer
C: Cyclophosphamide
CI: Confidence interval
CMF: Cyclophosphamide, methotrexate and fluorouracil
DBCG: Danish Breast Cancer Group
DFS: Disease-free survival
EBCTCG: Early Breast Cancer Trialists’ Collaborative Group
ER: Estrogen receptor
FFPE: Formalin-fixed, paraffin-embedded blocks
Her2: Human epidermal growth factor receptor 2
Her2-E: Human epidermal growth factor receptor 2-enriched
HR: Hazard ratios
IHC: Immunohistochemistry
OS: Overall survival
PAM50: Prediction analysis of microarray 50
ROR: Risk of recurrence

## References

[1] Bonadonna G, Brusamolino E, Valagussa P, Rossi A, Brugnatelli L, Brambilla C (1976). Combination chemotherapy as an adjuvant treatment in operable breast cancer. N Engl J Med.

[2] Ejlertsen B, Mouridsen HT, Jensen MB, Andersen J, Andersson M, Kamby C (2010). Cyclophosphamide, methotrexate, and fluorouracil; oral cyclophosphamide; levamisole; or no adjuvant therapy for patients with high-risk, premenopausal breast cancer. Cancer.

[3] Anonymous (1992). Adjuvant chemotherapy of breast cancer. NIH consensus development conference, July 14–16, 1980. Cancer Treat Res.

[4] Early Breast Cancer Trialists’ Collaborative Group (EBCTCG) (2005). Effects of chemotherapy and hormonal therapy for early breast cancer on recurrence and 15-year survival: an overview of the randomised trials. Lancet.

[5] Peto R, Davies C, Godwin J, Gray R, Pan HC, Clarke M (2012). Comparisons between different polychemotherapy regimens for early breast cancer: meta-analyses of long-term outcome among 100,000 women in 123 randomised trials. Lancet.

[6] Perou CM, Sorlie T, Eisen MB, van de Rijn M, Jeffrey SS, Rees CA (2000). Molecular portraits of human breast tumours. Nature.

[7] Cheang MC, Voduc KD, Tu D, Jiang S, Leung S, Chia SK (2012). Responsiveness of intrinsic subtypes to adjuvant anthracycline substitution in the NCIC.CTG MA.5 randomized trial. Clin Cancer Res.

[8] Albain KS, Barlow WE, Shak S, Hortobagyi GN, Livingston RB, Yeh IT (2010). Prognostic and predictive value of the 21-gene recurrence score assay in postmenopausal women with node-positive, oestrogen-receptor-positive breast cancer on chemotherapy: a retrospective analysis of a randomised trial. Lancet Oncol.

[9] Wallden B, Storhoff J, Nielsen T, Dowidar N, Schaper C, Ferree S (2015). Development and verification of the PAM50-based Prosigna breast cancer gene signature assay. BMC Med Genet.

[10] Gnant M, Filipits M, Greil R, Stoeger H, Rudas M, Bago-Horvath Z (2014). Austrian Breast and Colorectal Cancer Study Group. Predicting distant recurrence in receptor-positive breast cancer patients with limited clinicopathological risk: using the PAM50 risk of recurrence score in 1478 postmenopausal patients of the ABCSG-8 trial treated with adjuvant endocrine therapy alone. Ann Oncol.

[11] Nielsen TO, Jensen MB, Burugu S, Gao D, Joergensen CL, Balslev E (2017). High-risk premenopausal luminal a breast cancer patients derive no benefit from adjuvant cyclophosphamide-based chemotherapy: results from the DBCG77B clinical trial. Clin Cancer Res.

[12] Ejlertsen B, Mouridsen HT, Jensen MB, Andersen J, Cold S, Edlund P (2007). Improved outcome from substituting methotrexate with epirubicin: results from a randomised comparison of CMF versus CEF in patients with primary breast cancer. Eur J Cancer.

[13] Knoop AS, Knudsen H, Balslev E, Rasmussen BB, Overgaard J, Nielsen KV (2005). Retrospective analysis of topoisomerase IIa amplifications and deletions as predictive markers in primary breast cancer patients randomly assigned to cyclophosphamide, methotrexate, and fluorouracil or cyclophosphamide, epirubicin, and fluorouracil: Danish breast Cancer cooperative group. J Clin Oncol.

[14] O’Malley FP, Chia S, Tu D, Shepherd LE, Levine MN, Bramwell VH (2009). Topoisomerase II alpha and responsiveness of breast cancer to adjuvant chemotherapy. J Natl Cancer Inst.

[15] Di Leo A, Desmedt C, Bartlett JM, Piette F, Ejlertsen B, Pritchard KI (2011). HER2/TOP2A meta-analysis study group. HER2 and TOP2A as predictive markers for anthracycline-containing chemotherapy regimens as adjuvant treatment of breast cancer: a meta-analysis of individual patient data. Lancet Oncol..

[16] Ejlertsen B, Tuxen MK, Jakobsen EH, Jensen MB, Knoop AS, Hoejris I (2017). Adjuvant cyclophosphamide and docetaxel with or without epirubicin for early TOP2A-normal breast cancer: DBCG 07-READ, an open-label, phase III, randomized trial. J Clin Oncol.

[17] Blum JL, Flynn PJ, Yothers G, Asmar L, Geyer CE, Jacobs SA (2017). Anthracyclines in early breast cancer: the ABC trials–USOR 06-090, NSABP B-46-I/USOR 07132, and NSABP B-49 (NRG oncology). J Clin Oncol.

[18] Joergensen CL, Nielsen TO, Bjerre KD, Liu S, Wallden B, Balslev E (2014). PAM50 breast cancer intrinsic subtypes and effect of gemcitabine in advanced breast cancer patients. Acta Oncol.

[19] Tutt A, Ellis P, Kilburn L, Gilett C, Pinder S, Abraham J, et al. TNT: A randomized phase III trial of carboplatin compared with docetaxel for patients with metastatic or recurrent locally advanced triple negative or BRCA1/2 breast cancer. 2014 San Antonio Breast Cancer Symposium. Abstract S3-01. Presented December 11, 2014.

[20] Gluz O, Liedtke C, Huober J, Peyro-Saint-Paul H, Kates RE, Kreipe HH (2016). Comparison of prognostic and predictive impact of genomic or central grade and immunohistochemical subtypes or IHC4 in HR+/HER2- early breast cancer: WSG-AGO EC-doc trial. Ann Oncol.

[21] Nielsen TO, Parker JS, Leung S, Voduc D, Ebbert M, Vickery T (2010). A comparison of PAM50 intrinsic subtyping with immunohistochemistry and clinical prognostic factors in tamoxifen-treated estrogen receptor-positive breast cancer. Clin Cancer Res.

[22] Laenkholm, AV, Jensen MB, Eriksen JO, Kiboell T, Rasmussen BB, Knoop AS, et al. Prediction of 10yr distant recurrence (DR) using the Prosigna (PAM50) assay in a Danish Breast Cancer Cooperative Group (DBCG) cohort of postmenopausal Danish women with hormone receptor-positive (HR+) early breast cancer (EBC) allocated to 5 yr of endocrine therapy (ET) alone. J Clin Oncol 2015; 33(suppl; abstr 546).

[23] Altman DG, McShane LM, Sauerbrei W, Taube SE (2012). Reporting recommendations for tumor marker prognostic studies (REMARK): explanation and elaboration. PLoS Med.

[24] Nielsen T, Wallden B, Schaper C, Ferree S, Liu S, Gao D (2014). Analytical validation of the PAM50-based Prosigna breast Cancer prognostic gene signature assay and nCounter analysis system using formalin-fixed paraffin-embedded breast tumor specimens. BMC Cancer.

