# Supplementary figures and images for: The Prosigna gene expression assay and responsiveness to adjuvant cyclophosphamide-based chemotherapy in premenopausal high-risk patients with breast cancer
Source: Breast Cancer Res. 2018 Jul 27;20:79. doi: 10.1186/s13058-018-1012-0 (PMC6062869; doi:10.1186/s13058-018-1012-0)

Figure S1

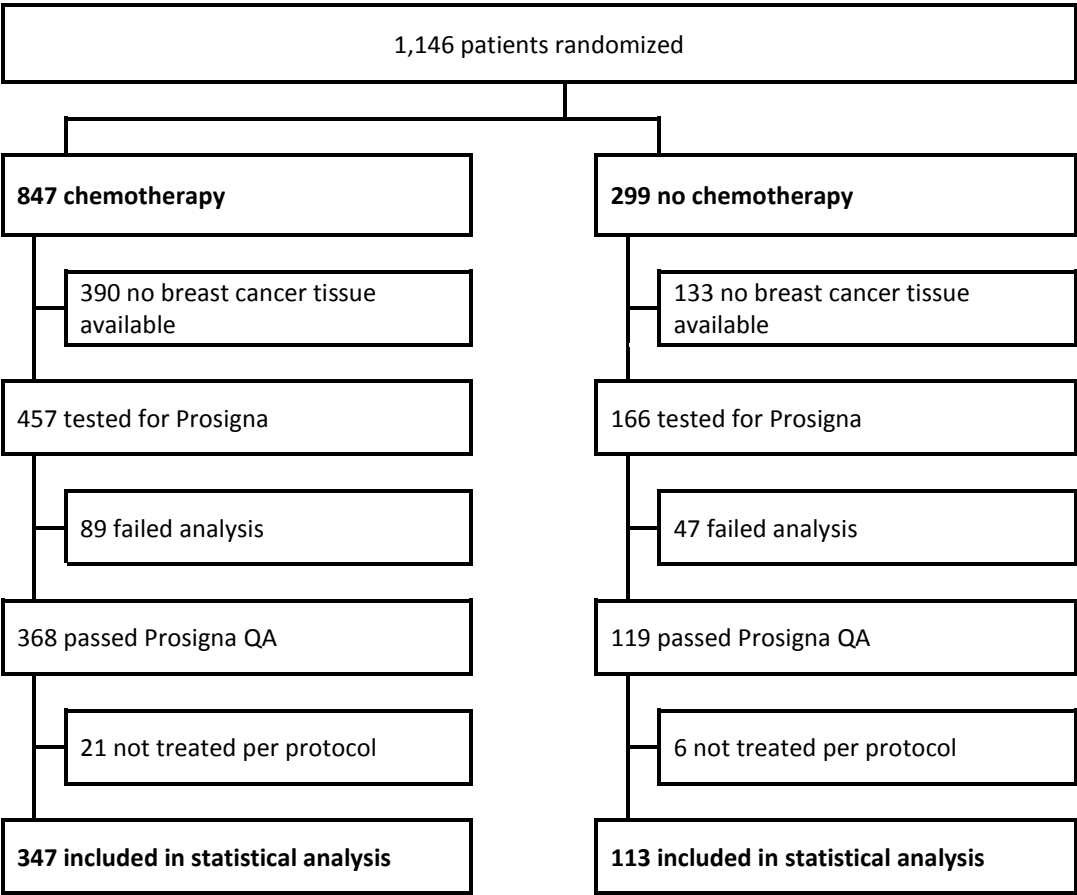

Supplement: Supplementary file 1 — Figure S1. Consort flow diagram. (PDF 234 kb) [file 13058_2018_1012_MOESM1_ESM.pdf]
